# Preparedness of final year medical students in caring for lesbian, gay, bisexual, and transgender patients with mental illness

**DOI:** 10.4102/sajpsychiatry.v29i0.1998

**Published:** 2023-04-28

**Authors:** Ahmed Badat, Sanushka Moodley, Laila Paruk

**Affiliations:** 1 Department of Psychiatry, Faculty of Health Sciences, University of the Witwatersrand, Johannesburg, South Africa

**Keywords:** LGBT, psychiatry, medical education, South African medical students, stigma, the lesbian gay bisexual and transgender development of clinical skills scale

## Abstract

**Background:**

Lesbian, gay, bisexual, and transgender (LGBT) individuals have a higher prevalence of mental illness compared to the general population. Discriminatory behaviour from mental health care providers impedes access to culturally competent mental health care. Undergraduate psychiatry education plays an important role in adequately preparing medical doctors to care for mental illness in LGBT patients.

**Aim:**

This study aims to assess the knowledge, attitudes and clinical preparedness of final-year medical students in caring for LGBT patients after completion of their psychiatry rotation.

**Setting:**

Faculty of health sciences at a large public university in Gauteng.

**Methods:**

This was a cross-sectional study using an anonymous self-administered questionnaire. The questionnaire comprised demographic data, the lesbian, gay, bisexual, and transgender development of clinical skills scale (LGBT-DOCSS) and questions relating to their subjective knowledge and preparedness in LGBT mental health care. The LGBT-DOCSS is a validated tool consisting of three subscales: basic knowledge, attitudinal awareness, and clinical preparedness.

**Results:**

Data from 170 final-year students were used in the analyses. Participants scored within the low range for clinical preparedness and basic knowledge subscales but high in the attitudinal subscale. Gender, sexual orientation and academic background were associated with higher overall scores and higher basic knowledge and attitudinal awareness scores.

**Conclusion:**

Final-year medical students were not adequately prepared in caring for LGBT patients with mental illness as indicated by the LGBT-DOCSS.

**Contribution:**

This study identifies a gap in undergraduate psychiatric training in providing culturally competent mental health care for a vulnerable population.

## Introduction

The lesbian, gay, bisexual, and transgender (LGBT) population are a diverse population group with unique health care needs. While each subgroup of the population consists of individuals of different race groups, ages, socioeconomic status, they share stigma and discrimination from the health care system and society at large.^1,2,3,4^ South Africa is considered as one of the most progressive countries in Africa in terms of LGBT rights, as the first African country to legalise same-sex marriage.^5^ Additionally, the South African Constitution includes provisions that prohibit discrimination on the basis of sexual orientation.^5^ The South African Bill of Rights, section 27, states that all citizens are allowed the right of access to health care services without discrimination based on their sexual minority status.^5,6^

Despite the efforts to provide equal access to health care for the lesbian, gay, bisexual, and transgender population in South Africa, discrimination towards this group remains prevalent.^4,6^ Homophobia, defined as an attitude of hostility towards an individual based on their sexual orientation, and transphobia, the discrimination of individuals who do not conform to societal gender expectations, contribute to this discrimination.^7,8^ This is often expressed through prejudice, harassment and violence towards transgender individuals.^8^ A study conducted in 2008 aimed to understand the interactions between gay men, non-gay identifying men who have sex with men (MSM) and public health care workers. The study found that access to non-stigmatising health care for these populations is limited, with openly identifying gay men experiencing more verbal harassment from health care workers.^9^

Lesbian and bisexual women also reported discrimination when accessing health care in South Africa. The double marginalisation faced by these women because of their gender and sexual minority status perpetuates neglect by the health care system.^10^ Research conducted by Müller in South Africa shows that all transgender participants experienced discrimination by health care workers on the basis of their gender identity and/or sexual orientation.^4^ Studies have shown that sexual and gender minorities face neglect and discrimination by health care workers based on religious, moral and political beliefs.^9^ As a result, accessing health care for the LGBT population in South Africa is challenging.^4^ There is increasing evidence supporting affirmative mental health practices for sexual and gender minorities, which acknowledge the multiple barriers to care at both structural and individual levels for these populations.^11^

Research suggests that LGBT individuals may have a higher prevalence of certain mental health conditions compared to the general population.^12^ Studies have found that LGBT individuals may be at an increased risk for depression, anxiety and suicide, as well as substance abuse and post-traumatic stress disorder (PTSD) when compared to the general population.^12^ A systematic review found an increased prevalence of depression, anxiety disorders, suicidal ideation and substance use disorders among sexual and gender minority populations in comparison to the heterosexual population.^11^ There is limited research on the prevalence of mental illness in the South African LGBT population.^6^ Lesbian, gay, bisexual, and transgender individuals may be undercounted in surveys and data collections because of discomfort with disclosing their sexual orientation or gender identity.^11^

The Minority Stress Theory is a popular explanation for the higher rates of mental illness in the LGBT population.^13^ It suggests that additional stressors experienced by stigmatised populations may impact their physical and mental health, leading to health disparities in the LGBT community.^14^ Researchers propose a model of proximal and distal stressors, including internalised homophobia, expectations of negative outcomes because of perceived stigma, discrimination, institutionalised discrimination, victimisation and prejudice.^15^ Structural and institutional discrimination are systemic biases and barriers in laws, policies and social institutions that limit the rights and opportunities of specific groups, including LGBT individuals. These discriminatory practices lead to unequal access to resources and services, such as health care, and perpetuate marginalisation. The Minority Stress Theory explains how the increased prevalence of mental illness in the LGBT population is a result of the additional stressors from societal prejudice and internalised negative attitude.^13,15,16,17^

Accessing health care, especially mental health care, is crucial for the LGBT population as they have a higher prevalence of mental illnesses.^11,18,19^ However, negative attitudes of health care workers can create barriers to accessing health care. Therefore, undergraduate medical education in psychiatry should prepare future clinicians to provide adequate care for LGBT individuals seeking mental health care.^6^

A systematic review of clinicians’ competency in caring for LGBT patients revealed a need for reinforced training in this area. The lack of inclusion of sexual and gender minority health in health care workers’ training contributes to neglect of LGBT issues.^20^ Research shows that medical students with more exposure to LGBT patients and topics are more competent in assessing their unique health care needs.^21,22,23^ A scoping review of interventions to improve health care for LGBT patients in South Africa found that the lack of inclusion of sexual and gender minority health in the training of health care workers contributes to the neglect of LGBT issues.^6^ Undergraduate medical education is crucial in preparing future doctors to address the unique health care needs of the LGBT population, particularly in mental health care.^21,22,23^ This study evaluates the competency of medical students in addressing these needs, identifies gaps in their training and suggests areas for improvement. It aims to provide insights into how undergraduate medical education can better equip future doctors to provide non-judgmental care for LGBT patients, particularly those with mental health issues, addressing health disparities and ensuring equal care for all patients.

### Aims

- To assess the knowledge, attitudes and clinical preparedness of final-year medical students in caring for LGBT patients after the completion of their psychiatry rotation.
- To evaluate the level of preparedness of students in caring for mental illness among LGBT patients.

### Objectives

- To conduct a survey among final-year medical students to measure their knowledge, attitudes and clinical preparedness in caring for LGBT patients using the LGBT-DOCSS.
- To compare the demographic data (age, gender, sexual orientation, religious affiliation and academic background) of the participants in relation to their level of preparedness in caring for LGBT patients with mental illness.

## Research methods and design

### Study design

This is a cross-sectional, quantitative, descriptive study.

### Method

To use the lesbian, gay, bisexual, and transgender development of clinical skills scale (LGBT-DOCSS) and questionnaire relating to mental health in the LGBT population to conduct a survey among final-year medical students.^24^ A questionnaire requesting demographic data was also used.

### Setting

The study was conducted at the University of the Witwatersrand.

### Study population

Final-year medical students enrolled in 2021 at the University of the Witwatersrand, comprising students with diverse demographic characteristics (age, gender, sexual orientation, religious affiliation and academic background). All consenting students who had completed their psychiatry rotation were included in the study. Some students had enrolled in the programme via the Graduate Entry Medical Programme (GEMP) after completion of another bachelor’s degree or higher qualification, while the others were enrolled into the Bachelor of Medicine and Bachelor of Science degree after completing high school. The final-year students had completed their 6-week academic rotation at dedicated psychiatric units. This involved formal lectures, smaller group tutorials, after-hour calls and clinical exposure to patients. There is no stipulated teaching regarding LGBT mental health in the formal academic rotation, but clinical exposure to LGBT patients may differ between students.

### Sample

Non-probability convenience sample of 84 students, selected from a class of 325 final-year medical students, with 170 students completing the questionnaire.

### Data collection

The study surveyed students using the LGBT-DOCSS tool, designed by Bidell to assess clinical development in caring for LGBT patients.^24^ The tool includes 18 items across three subscales, namely clinical preparedness, attitudinal awareness, and basic knowledge, with higher scores indicating greater preparedness. The term ‘development’ was chosen over ‘competency’ to emphasise the ongoing process of obtaining cultural competence. The tool uses a 7-point Likert scale, with eight items reverse-scored. Scoring instructions are not provided to participants.^24^

The LGBT-DOCSS was developed with the goal of having sound reliability and validity, as demonstrated through comparison to other existing scales such as the Genderism and Transphobia Scale-Revised-Short-Form (GTS-R-SF) and the Marlow-Crowne Social Desirability Short Form-A (MCSD-A).^24^ The scale was created using a sample of general medical practitioners, clinical psychologists and medical and psychology students from the United States and United Kingdom and has demonstrated good internal consistency, test-retest reliability and content discrimination validity.^24^ However, the authors caution that the LGBT-DOCSS should not be used as a comprehensive measure of LGBT cultural competence, as it only measures explicit bias and does not assess internal biases, which also impact clinical preparedness. Permission to use the questionnaire is not required from the developer.^24^

This study also included a supplementary questionnaire to gauge the participants’ knowledge and comfort level regarding mental illness and psychosocial stressors affecting the LGBT population. Participants were asked to rate their understanding of these topics, as well as their confidence in collecting a sexual and relationship history from LGBT patients and providing care for LGBT patients with mental illness. This was titled Mental Health in the LGBT Population. Demographic information such as age, gender, sexual orientation, religious affiliation and academic background (graduate or otherwise) was also collected through a demographic questionnaire.

Questionnaires were given to participants after they took a multiple-choice psychiatry exam to avoid any obligation to participate and to maintain confidentiality and reduce anxiety related to the exam. Participants were instructed to deposit their completed questionnaires in designated boxes at the exam venue.

### Data analysis

The data collected from the questionnaires were analysed using Microsoft Excel and the R software (version 4.00). A Shapiro-Wilk test was performed to check for normality and Q-Q plots were used to visualise the data. Non-parametric methods were applied to data that deviated from normality or consisted of categorical variables.

Chi-squared goodness of fit test was used to analyse the relationship between single categorical variables and deviation from chance. For comparison between two categorical variables, chi-squared tests of association were employed. Adjusted residual values were calculated to determine which combinations between categorical variables had a significant effect on the model outcomes.

The frequency of scores was used to assess the most common score categories among the study population for the total scores obtained on various subscales, including LGBT-DOCSS, clinical preparedness and knowledge of mental health conditions. Spearman rank correlations were used to examine the association between continuous variables. Differences in scores for multiple factors were analysed using Mann-Whitney U tests and Kruskal-Wallis tests. In case of significant results in the Kruskal-Wallis tests, Dunn’s post-hoc tests were used to identify specific differences.

The data were presented descriptively, including mean and standard deviation for continuous data and count and percentages for categorical data. The data were presented in various forms, including tables, charts and text.

### Ethical considerations

Permission to conduct this study was obtained from the Deputy Registrar, the Head of Department of Psychiatry and the Unit for Undergraduate Medical Education (UUME) at the University of the Witwatersrand. Ethics clearance (M210225) was also obtained from the research review board at a large public university in Gauteng.

## Results

### General characteristics

A total of 170 students at the University of the Witwatersrand participated in the study. The average age of the participants was 25.3 years with a standard deviation of 2.9. A higher proportion of the participants were female, identified as heterosexual, and affiliated with Christianity (as shown in Table 1). The number of graduate and non-graduate participants did not show a significant difference.

**TABLE 1:** Characteristics of participants in the study.

Variables	Count	Percent	Statistics†		
			χ^2^	*df*	*p*
**Gender**	-	-	**4.61** ‡	**1** ‡	**0.031** ‡
Female	99	58.2	**-**	**-**	**-**
Male	71	41.8	**-**	**-**	**-**
**Sexual orientation**	**-**	**-**	**245.87** ‡	**2** ‡	**< 0.001** ‡
Heterosexual	153	90.0	**-**	**-**	**-**
Homosexual	6	3.5	**-**	**-**	**-**
Other	11	6.5	**-**	**-**	**-**
**Religion**	-	-	**219.14** ‡	**6** ‡	**< 0.001** ‡
Atheist	19	11.2	-	-	-
Christian	90	53.3	-	-	-
Hindu	14	8.3	-	-	-
Jewish	13	7.7	-	-	-
Muslim	22	12.4	-	-	-
Non-affiliated	10	5.9	-	-	-
Other	2	1.2	-	-	-
**Academic background**	-	-	2.84	1	0.091
Graduate	74	43.5	-	-	-
Non-graduate	96	56.5	-	-	-

Note: Count and percentage data are shown. Statistics, chi-squared tests per variable; significant outcomes are shown in bold.

*df*, degree of freedom.

† , chi-squared tests per variable;

‡ , significant outcomes.

### Total scores obtained on the lesbian, gay, bisexual, and transgender development of clinical skills scale and mental health in the lesbian, gay, bisexual, and transgender questionnaires

The medians (1st and 3rd interquartiles) were calculated for the total score obtained on the LGBT-DOCSS and Mental Health in the LGBT Population questionnaires separately. For the LGBT-DOCSS questionnaire, the total median was 3.28. The medians of the clinical preparedness, attitudinal awareness and basic knowledge subscales were 3.28, 6.57 and 4.50, respectively. Participants scored highest on the attitudinal awareness subscale and lowest on the clinical preparedness subscale. A median score of 4.2 was calculated for the Mental Health in the LGBT Population questionnaire.

**FIGURE 1:**
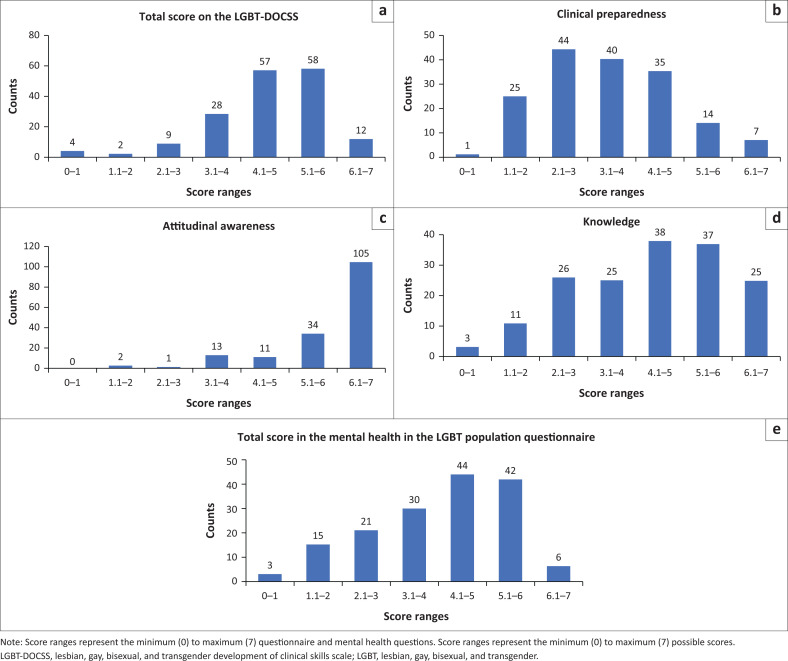
Frequency histograms showing the distribution of: (a) total score on the LGBT-DOCSS, (b) clinical preparedness, (c) attitudinal awareness, (d) basic knowledge subscales obtained from the LGBT-DOCSS questionnaire and (e) the total score in the mental health LGBT population questionnaire.

Figure 1 shows the frequency plots of scores for the total score obtained on the LGBT-DOCSS with its subscales and the Mental Health in the LGBT Population questionnaire. Significantly more participants scored in the 4 to 6 range for the total score obtained on the LGBT-DOCSS (χ^2^ = 144.67, *df* = 6, *p* < 0.001) (Figure 1). For the clinical preparedness subscale, significantly more participants scored in the 2 to 4 range (χ^2^ = 71.49, *df* = 6, *p* < 0.001). A significant majority of the participants scored in the 6 to 7 range for the attitudinal awareness subscale (χ^2^ = 360.09, *df* = 6, *p* < 0.001). Significantly more participants scored in the 2 to 4 range for the basic knowledge subscale (χ^2^ = 41.56, *df* = 6, *p* < 0.001). Finally, for the mental health questionnaire, significantly more participants scored in the 4 to 6 range (χ^2^ = 69.91, *df* = 6, *p* < 0.001) (Figure 1).

### Association between demographic data and questionnaire scores

#### Age of participants

The results showed no significant correlation between the participants’ age and their scores on the LGBT-DOCSS (*r* = -0.14, *p* = 0.081) or the Mental Health in the LGBT Population questionnaire. Further analysis indicated a lack of significant relationship between age and scores on the clinical preparedness (*r* = -0.06, *p* = 0.439), attitudinal awareness (*r* = -0.09, *p* = 0.256) and basic knowledge (*r* = -0.02, *p* = 0.815) subscales of the LGBT-DOCSS questionnaire.

#### Gender of participants

Gender had a significant impact on the total score of the LGBT-DOCSS questionnaire (*U* = 2702.5, *n* = 99, *p* = 0.031). Female participants had significantly higher median scores than males for total scores on LGBT-DOCSS, as well as on the attitudinal awareness (*U* = 2220, *n* = 99, *p* < 0.001) and basic knowledge (*U* = 2220, *n* = 99, *p* < 0.001) subscales. However, gender was not found to be a significant predictor of scores on the clinical preparedness subscale (*U* = 3270, *n* = 99, *p* = 0.768) or the Mental Health in the LGBT population questionnaire (*U* = 2745.5, *n* = 99, *p* = 0.142).

#### Sexual orientation of participants

Sexual orientation was a significant predictor of the total (*U* = 755, *p* = 0.005), knowledge subscale (*U* = 830.5, *p*-value = 0.050) and mental health questionnaire (*W* = 521.5, *p* = 0.002) scores. For these variables, sexual minority respondents (a combination of students who identified as homosexual or other) had significantly greater median scores than those identifying as heterosexual. Sexual orientation was not a significant predictor of the clinical preparedness subscale (*W* = 1008.5, *p* = 0.170) and attitudes subscale (*W* = 959, *p* = 0.010) scores.

#### Religious affiliation of participants

The results showed that religious affiliation was not a significant predictor of the scores obtained on the LGBT-DOCSS questionnaire (*U* = 1699.00 [29, 137], *p* = 0.224) or the Mental Health in the LGBT population questionnaire (*U* = 1524.00 [28, 131], *p* = 0.189). There were also no significant differences in scores obtained on the clinical preparedness (*U* = 1872.00 [29, 136], *p* = 0.672), attitudinal awareness (*U* = 1763.00 [29, 136], *p* = 0.374) or basic knowledge (*U* = 1793.50 [29, 135], *p* = 0.482) subscales of the LGBT-DOCSS.

#### Academic background of participants

The academic background of respondents was a significant predictor of basic knowledge subscale scores only (*U* = 219, *n* = 73, *p* = 0.025). Graduate students scored significantly higher median scores than non-graduate medical students for the basic knowledge subscale scores. The academic background of participants was not a significant predictor of total scores on the LGBT-DOCSS (*U* = 401, *n* = 73, 96, *p* = 0.494), attitudinal awareness subscale (*U* = 359.5, *n* = 73, 96, *p* = 0.585), clinical preparedness subscale (*U* = 439.5, *n* = 73, 96, *p* = 0.594) and Mental Health in the LGBT Population questionnaire (*U* = 354.5m *n* = 73, 96, *p* = 0.324) scores.

### Participants’ responses regarding if they felt prepared in caring for lesbian, gay, bisexual, and transgender patients with mental illness after completion of their psychiatry rotation

The participants’ level of preparedness in caring for LGBT patients with mental illness was evaluated through a 7-point Likert scale questionnaire, where they indicated their agreement with the statement ‘My psychiatry rotations have adequately prepared me in caring for mental illness in LGBT patients’. Their responses were analysed in relation to their sociodemographic profile and the scores they received on the LGBT-DOCSS and its sub-scales: (1) clinical preparedness, (2) attitudinal awareness and (3) basic knowledge.

#### Sociodemographic variables and response to the statement

There was no correlation between the age of the participants and responses to the Likert scores for the statement (Spearman *r* = -0.01, *p* = 0.934).

**FIGURE 2:**
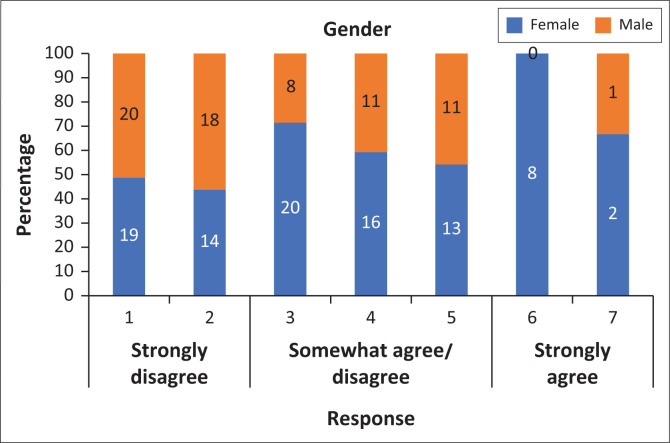
The percentage of participants according to gender different to a 7-point Likert score for the statement ‘My psychiatry rotations have adequately prepared me in caring for mental illness in lesbian, gay, bisexual and transgender (LGBT) patients’.

Gender was a significant predictor of the responses (χ^2^ = 18.26, *df* = 6, *p* = 0.001; Figure 2). Specifically, female-identifying participants tended to strongly agree with the statement, whereas students who identified as male tended to strongly disagree with the statement. The remaining variables such as sexual orientation (χ^2^ = 17.80, *df* = 12, *p* = 0.122), religious affiliation (χ^2^ = 47.46, *df* = 36, *p* = 0.100) and academic background (χ^2^ = 4.17, *df* = 6, *p* = 0.654) did not show a statistically significant difference.

#### Scores obtained on lesbian, gay, bisexual, and transgender development of clinical skills scale and response to the statement

The relationship between the LGBT-DOCSS scores and the statement ‘My psychiatry rotations have adequately prepared me in caring for mental illness in LGBT patients’ was significant and positive. The total scores obtained on the LGBT-DOCSS had a significant positive correlation with the statement (Spearman’s rho = 0.42, *p* < 0.001), which indicates that an increase in total scores on the LGBT-DOCSS was associated with a tendency to agree with the statement.

Additionally, the clinical preparedness subscale scores were positively correlated with the statement (Spearman’s rho = 0.55, *p* < 0.001), with higher scores on the clinical preparedness score being linked to a greater tendency to agree with the statement (as shown in Figure 2). The basic knowledge subscale scores also showed a positive correlation with the statement (Spearman’s rho = 0.27, *p* < 0.001), suggesting that respondents with higher knowledge scores were more likely to agree with the statement. Contrarily, the attitudinal awareness subscale scores showed no correlation with the statement (Spearman’s rho = 0.07, *p* = 0.404).

## Discussion

This study assessed final-year medical students’ competency in addressing mental health needs of LGBT patients using the LGBT-DOCSS and a supplementary questionnaire. In the absence of proposed cut-off scores by the developer of the LGBT-DOCSS, this study considered total scores greater than six as high competency, scores between five and six as moderate competency and scores less than five as low competency, based on previous studies.^25^ Final-year medical students showed low competency in caring for LGBT patients with mental illness, according to their scores on the LGBT-DOCSS, which were in line with previous research.^25,26^ Their scores on the clinical preparedness and basic knowledge subscales were also in the low competency range. To address this issue, it is crucial to enhance training and clinical exposure to LGBT patients during undergraduate psychiatry training in South Africa. Medical students should have a comprehensive understanding of key mental health topics relevant to the LGBT population, including discrimination, body image, substance use disorders, coming out and stigma.^27^ Preclinical psychiatry training can empower students to effectively engage in psychiatric interviews with LGBT patients by equipping them with the knowledge and skills needed to discuss mental health issues such as gender identity and preferred pronouns.^27^

Medical students in this study demonstrated high attitudinal awareness but low competency in total score, clinical competency and basic knowledge subscales. This pattern has been seen in other studies and highlights the need for increased cultural competency in LGBT health care for medical students in South Africa.^26^ Despite the encouraging result, patients in South Africa have reported experiences of prejudice and homophobic behaviour from health care practitioners.^9^

Female participants in the study scored higher on the LGBT-DOCSS total score, as well as the attitudinal awareness and basic knowledge subscales compared to male participants. This aligns with previous research showing that female practitioners demonstrate greater empathy and communication skills when compared to male practitioners.^28^ Previous research has found that female practitioners have better communication skills, are more likely to build partnerships, and ask more psychosocial questions than male practitioners. They also tend to hold a more continuous view of gender compared to males who have a more binary perspective, according to a systematic review by Howick et al.^28,29^ Female participants also scored higher in basic knowledge and attitudinal awareness, possibly because of their more positive attitude towards LGBT patients. More female participants felt adequately prepared to care for mental illness in LGBT patients than male participants. However, the role of gender in providing health care to LGBT patients needs further research.^30^

Sexual minority students demonstrated higher knowledge and self-reported preparedness for LGBT patient care than heterosexual peers in this study, possibly because of a greater personal investment in understanding the population’s need. The stigma and discrimination faced by the LGBT community may also drive them to educate themselves on relevant issues and advocate for their health. However, further research is needed to confirm reasons for the difference in scores. The data on the experiences of LGBT medical students in South Africa is limited, but previous studies have shown that sexual and gender minority medical students are more likely to advocate for research and clinical programmes that address their health needs and choose specialities that they perceive as being more inclusive.^31,32^ Sexual orientation was not a reliable predictor of better knowledge in LGBT health care, emphasising the need for improved training in this area for all medical students.^33^

Religious affiliation did not have a significant impact on the study’s outcomes, which contrasts with past research showing a link between religious affiliation and negative attitudes towards LGBT individuals.^32,33^ Religious beliefs have been used to explain homophobia among health care practitioners in South Africa.^6^

Graduate students scored higher on the basic knowledge subscale, indicating previous exposure to LGBT patients or education contributed to higher scores. However, academic background was not a significant predictor of feeling prepared to care for LGBT mental illness, suggesting learning gaps in psychiatry curricula.

### Limitations

Limitations include the study’s restricted focus on final-year medical students at a specific institution in South Africa, uncertain validity of the LGBT-DOCSS in the country, potential heterosexist bias and response bias and limitations in assessing competence in caring for various sexual and gender minorities. Heterosexist bias, which occurs when experiences are defined in strictly heterosexual terms without considering sexual and gender minorities.^34^ Finally, the LGBT-DOCSS does not evaluate competence in caring for queer, intersex and other sexual and gender minorities.

## Conclusion

This study emphasises the necessity for better training of medical students in caring for LGBT patients with mental illness, as shown by scores on the LGBT-DOCSS. Although students have positive attitudes towards LGBT patients, their scores on basic knowledge and clinical preparedness imply that gaps in their training must be addressed. More culturally competent LGBT medical education is required in undergraduate psychiatry programmes. Further research should be conducted to identify effective methods of delivering this education and assessing its impact on the quality of care provided to LGBT patients. Expanding the study to other medical schools in South Africa would provide a clearer understanding of the state of LGBT medical education in the country and inform the development of more effective training programmes for future health care providers.
